# Comparison of Dietary Supplementation with Krill Oil, Fish Oil, and Astaxanthin on an Experimental Ethanol-Induced Gastric Ulcer Model: A Biochemical and Histological Study

**DOI:** 10.3390/nu16203426

**Published:** 2024-10-10

**Authors:** Esra Tansu Sarıyer, Murat Baş, Hatice Çolak, Naziye Özkan Yenal, Özlem Unay Demirel, Meral Yüksel

**Affiliations:** 1 Department of Nutrition and Dietetics, Institute of Health Sciences, Acibadem Mehmet Ali Aydinlar University, 34752 Istanbul, Turkey; 2 Department of Nutrition and Dietetics, Faculty of Health Science, University of Health Sciences, 34668 Istanbul, Turkey; 3 Department of Nutrition and Dietetics, Faculty of Health Sciences, Acibadem Mehmet Ali Aydinlar University, 34752 Istanbul, Turkey; murat.bas@acibadem.edu.tr; 4 Department of Nutrition and Dietetics, Faculty of Health Sciences, Üsküdar University, 34662 Istanbul, Turkey; hatice.colak@uskudar.edu.tr; 5 Department of Pathology Laboratory Techniques, Vocational School of Health-Related Services, Marmara University, 34865 Istanbul, Turkey; nozkan@marmara.edu.tr; 6 Department of Medical Biochemistry, Bahçeşehir University Göztepe Medical Park Hospital Central Laboratory, Faculty of Medicine, Bahçeşehir University, 34353 Istanbul, Turkey; ozlemunay.demirel@medicalpark.com.tr; 7 Department of Medical Laboratory Techniques, Vocational School of Health-Related Services, Marmara University, 34865 Istanbul, Turkey; meralyuksel@marmara.edu.tr

**Keywords:** gastric ulcer, liver, krill oil, fish oil, astaxanthin, oxidative stress, antioxidant

## Abstract

Background/Objectives: Despite advances in ulcer treatment research, the search for new, safe, and effective strategies for preventing and treating ulcer diseases persists. Methods: In this study, the protective effects of dietary supplementation with krill oil (KO), fish oil (FO), and astaxanthin (ASX) on an ethanol-induced gastric ulcer model were compared during biochemical and histological observations. Sprague–Dawley (*n* = 64) rats randomly divided into four groups—normal control (vehicle), KO, FO, and ASX groups—received the supplements via the orogastric route at a rate of 2.5% (*v*/*w*) of their daily feed consumption for 4 weeks. Then, ulcer induction was performed with ethanol. Results: The ulcer group showed increased levels of malondialdehyde (MDA), chemiluminescence (CL), and myeloperoxidase (MPO) activity and decreased levels of glutathione in the gastric tissues. While KO, FO, and ASX supplementation decreased chemiluminescence levels in the ulcer group, only ASX supplementation decreased MDA levels and MPO activity. Conclusions: In conclusion, supplementation with KO or FO has a similar protective effect against ethanol-induced ulcer damage, as it inhibits ROS formation and reduces lipid peroxidation. However, ASX supplementation has a higher protective effect than KO or FO supplementations against experimental ethanol-induced gastric lesions in rats, as it inhibits ROS formation and reduces neutrophil infiltration and lipid peroxidation.

## 1. Introduction

Peptic ulcer disease, which includes both gastric and duodenal ulcers, markedly affects the global population and has been associated with high morbidity and mortality over the past century [1,2]. In gastric ulcers, the balance between protective factors—such as mucus, bicarbonate, prostaglandins (PG), and antioxidant enzymes—and aggravating factors—such as gastric acid, pepsin, Helicobacter pylori infection (*H. pylori*), nonsteroid anti-inflammatory drugs (*NSAIDs*), smoking, alcohol, and oxidative stress—is disrupted. Ulcerations and mucosal injuries result from this disruption [1,3]. Excessive consumption of alcohol negatively affects the gastrointestinal system. Alcohol-induced stomach ulcer formation is hypothesized to be caused by reduced PG synthesis, increased cyclooxygenase (COX), lipoxygenase (LOX), cytokines, and reactive oxygen species (ROS) [4,5]. High alcohol consumption generally weakens gastric mucosal defenses, leading to gastric mucosal injuries and resulting in conditions such as gastritis, gastric ulcers, and gastric cancer [6].

The pharmacological treatment of ulcers involves the use of antacids, proton pump inhibitors (PPIs), antibiotics, and H2 receptor blockers [7]. Although some pharmacological treatments (such as PPIs and H2 receptor blockers) for stomach ulcers do not completely heal the ulcers, their long-term and continued use has been reported to increase the risk of hypersensitivity, arrhythmia, hypomagnesemia, and gastric cancer [7,8,9]. Therefore, the search for new, safe, and effective strategies for ulcer prevention and treatment has gained importance [7]. Regulating nutrition and utilizing dietary supplements can also lead to symptom reduction. Consuming essential fatty acids through diet can decrease peptic ulcer incidence, and fresh rice oil reduces gastric ulceration in experimental animal models. When adjusting diets for cases of peptic ulcers, dietary supplements play a crucial role. Powerful antioxidants, such as alpha-lipoic acid and resveratrol, provide protective and therapeutic qualities against experimental ulcers [10,11]. Avoiding fatty foods that increase digestive juice is crucial for effective ulcer treatment. Nevertheless, healthy fats are necessary for cell repair and overall health, particularly for the immune system [12].

Krill oil (KO), extracted from small crustaceans (*Euphausia superba*), has attracted attention due to its fatty acid content and beneficial effects on health. It contains omega-3 (*n*-3) polyunsaturated fatty acids (PUFA), phospholipids, flavonoids, and astaxanthin (ASX), as well as various vitamins and minerals. Owing to its nutritional composition, KO possesses antioxidant and anti-inflammatory effects [13,14], which have resulted from its antioxidant elements, such as vitamin E, choline, and ASX [15]. KO supplementation reduced inflammatory levels of tumor necrosis factor-alpha (TNF-α) and interleukin-8 (IL-8) in in vitro investigations [16,17]. Like KO, fish oil (FO) is a rich dietary source of *n*-3 fatty acids. Omega-3 fatty acids, such as docosahexaenoic acid (DHA) and eicosapentaenoic acid (EPA), are found in seafood, such as fish and algae, and in plant sources, including flaxseed oil, canola oil, walnuts, and purslane. Numerous studies have demonstrated the protective effects of *n*-3 fatty acids against cardiovascular disease, cancer, diabetes, and inflammatory illnesses. Leukotrienes, thromboxanes, and PGs made from EPA and DHA have anti-inflammatory properties [18]. Notably, the bioavailability of EPA and DHA after the intake of KO and FO differs. Gene expression studies using experimental animal models suggested that FO supplementation increased cholesterol synthesis pathway molecules, and KO supplementation regulated more metabolic pathways, such as glucose, fatty acid, and lipid metabolism pathways [19].

Unlike FO, KO contains ASX, a xanthophyll carotenoid found in various marine animals and some microorganisms. ASX cannot be synthesized in the human body. Experimental studies have suggested that ASX has 100 times more antioxidant activity than alpha-tocopherol and is 10 times stronger than zeaxanthin, lutein, canthaxanthin, and zeaxanthin [20]. By reducing the synthesis of pro-inflammatory cytokines via the nuclear factor kappa B (NF-κB) pathway, ASX lowers ROS production, hence exhibiting antioxidant effects [21]. Current evidence in the literature suggests that ASX has therapeutic and preventive benefits for various acute and chronic conditions, including kidney, liver, gastrointestinal, and neurological diseases [22].

Based on this knowledge, this study was designed to compare the protective effects of nutritional supplementation with KO and FO on tissue integrity, oxidant–antioxidant status, and neutrophil infiltration in inflamed gastric tissue in a rat model of ethanol-induced ulcers. To investigate a possible superiority of ASX-containing KO over ASX-free FO, a group of ASX-only treatments was also added to the study. Similarly, the effects of KO, FO, and ASX supplementation on biochemical and histopathological parameters of liver tissue were analyzed and compared.

## 2. Materials and Methods

### 2.1. Animals and Drugs

Male Sprague–Dawley rats (300–350 g) were obtained from the Üsküdar University Experimental Research Unit (ÜSKÜDAB) and were kept in a room with controlled temperature (22 ± 2 °C), humidity (65–70%), and 12 h light–12 h dark cycles. Rats were fed standard pellet chow and water ad libitum. All study procedures were permitted by the Üsküdar University Animal Experiments Local Ethics Committee (ÜÜ-HADYEK) with approval number 2021-08.

Some characteristics of the nutritional supplements given to the experimental animals were considered following the literature. For this purpose, KO, produced with Aker Biomarine patented technology and with an EPA + DHA content of 250 mg, was used (Tabilaç, Istanbul, Turkey) [23,24,25]. The type of FO to be applied to the experimental animals was provided in triglyceride form, containing 250 mg EPA + DHA and being IFOS certified (Eczacıbaşı-Kampotu, Istanbul, Turkey) [26,27], and ASX was provided by a pharmaceutical company (Donemed, Istanbul, Turkey) [28].

### 2.2. Experimental Design

Rats (*n* = 64) were randomly divided into four groups. All rats received a standard diet. A preliminary study was conducted to calculate the dietary supplementations for the rats. The amount of food and water consumed by the rats was monitored for one week. At the end of the week, it was found that the rats consumed between 20–22 g of food. Based on the calculation that 2.5% of the daily food intake should be provided as dietary supplementation, it was decided to administer 0.10–0.13 mL of KO, FO, or ASX supplementation to rats weighing 200–250 g.

We administered vehicle, KO, FO, or ASX via the orogastric route to the rats at a rate of 2.5% (*v*/*w*) of their daily feed consumption for 4 weeks [29,30,31]. Experimental animals in each group fasted for 24 h after nutritional supplementation and were divided into two groups. The rats (*n* = 8 per group) received absolute ethanol (5 mL/kg) or saline (control group) orally via gavage [32]. All animals were euthanized 1 h after ethanol administration under anesthesia (ketamine 100 mg/kg and xylazine 10 mg/kg), after which trunk blood was collected. The stomach and liver tissues were removed and separated for biochemical and histopathological analyses. Until the analysis, separated serum and tissue samples were kept in a −80 °C deep freezer. At the end of the experimental study, we formed eight groups: control group (C), ulcer group (U), fish oil + control group (FO + C), fish oil + ulcer group (FO + U), krill oil + control group (KO + C), krill oil + ulcer group (KO + U), astaxanthin + control group (ASX + C) and astaxanthin + ulcer group (ASX + U).

### 2.3. Macroscopic Analysis of Gastric Tissue

The freshly excised stomachs were examined macroscopically to analyze hemorrhagic lesions in the glandular mucosa. Immediately after decapitation, the stomachs were dissected out and cut along a greater curvature, and the mucosa was rinsed with cold normal saline to remove any blood contaminants. The length (mm) of each lesion was measured (three petechiae were counted as 1 mm), summed per stomach, and expressed as an erosion index (Table 1) [31].

### 2.4. Histopathological Examination of Gastric and Hepatic Tissues

For light microscopic investigations, gastric and liver tissue specimens were fixed in 10% neutral-buffered formalin, dehydrated in alcohol series, cleared in xylene, and embedded in paraffin. Paraffin sections (5 μm) were stained with hematoxylin–eosin (H&E). Gastric and hepatic injuries were assessed and semi-quantitatively scored using a 0–3 scoring system (0: none, 1: mild, 2: moderate, 3: severe) according to the modified criteria from previous studies [33,34]. Microscopic scoring (Olympus BH 2, Tokyo, Japan) of the tissue samples was performed by an experienced histologist (NYÖ) blinded to the experimental groups.

### 2.5. Measurement of Gastric and Hepatic Malondialdehyde and Glutathione Levels

Malondialdehyde (MDA) levels were evaluated to determine lipid peroxidation levels in the stomach and liver tissues of the rats. The formed pink color via thiobarbituric acid treatment was measured at a 532 nm wavelength in a spectrophotometer (Beckman DU730 UV/Vis, Brea, CA, USA). The results were expressed as nmol/g tissue [35]. For the tissue glutathione (GSH) measurement, the yellow color formed via Ellman’s reagent was measured spectrophotometrically at a 412 nm wavelength. The results were expressed as µmol/g tissue [35].

### 2.6. Measurement of Gastric and Hepatic Myeloperoxidase Activity

Using the method described by Hillegass et al., tissue myeloperoxidase (MPO) activity was assessed. Tissue samples were homogenized (10% weight/volume) in 50 mM potassium phosphate buffer (PB, pH 6.0) containing 0.5% hexadecyltrimethylammonium bromide (HETAB) and ethylenediaminetetraacetic acid and centrifuged at 12,000 rpm at 4 °C. After centrifugation for approximately 10 min, 2.9 mL of reaction mixture comprising 50 mM PBS, o-dianisidine, and 20 mM H_2_O_2_ solution was added to a 37 °C water bath. Using a spectrophotometer, MPO enzyme activity was assessed at a 460 nm wavelength. The results were expressed as U/g tissue [36].

### 2.7. Measurement of Gastric and Hepatic Chemiluminescence Assays

The chemiluminescence (CL) method can be used to determine ROS through enhancers. Probes such as luminol and lucigenin, called “enhancers,” were used for ROS measurement. Luminol is selective for hydroxyl radicals, hydrogen peroxide (H_2_O_2_), hydroperoxyl, and hypochlorite, while lucigenin measures superoxide radicals. Luminol (0.2 mM) or lucigenin (0.2 mM) was added to the tubes that contained tissue samples and PBS + HEPES buffer (0.5 M phosphate buffered saline containing 0.02 M HEPES [4-(2- hydroxyethyl)-1-piperazine-ethanesulfonic acid]; pH 7.4) and measured in a luminometer (Junior LB9509, Berthold, Bad Wildbad, Germany) for 5 min at 1 min intervals. For determination of nitric oxide in gastric and liver tissue samples, the luminol sodium salt and H_2_O_2_ system were used. For this purpose, K_2_CO_3_, Desferal, H_2_O_2_, and luminol sodium salt were added to the test tubes containing the tissue sample and PBS + HEPES buffer and counted at the same time as explained above. To reveal the difference in peroxynitrite, carboxy-PTIO was added to the NO measurement medium, incubated for 10 min, and the measurement was repeated. The difference was calculated as a peroxynitrite value. At the end of the measurements, the tissues were removed from the counting tubes, the fluids were blotted on filter paper, and dry weights were taken. The area under the curve was calculated, and the results were expressed as relative light units per mg tissue samples [37,38,39].

### 2.8. Measurement of Serum Lipids and Liver Function Tests in Serum Samples

Lipid parameters, such as total cholesterol (TC) (mg/dL), triglyceride (TG) (mg/dL), low-density lipoprotein cholesterol (LDL-C) (mg/dL), and high-density lipoprotein cholesterol (HDL-C) (mg/dL), were determined in serum obtained from blood samples of experimental animals. To assess liver function, serum samples were tested for alanine aminotransferase (ALT) (U/L), aspartate aminotransferase AST (U/L), and alkaline phosphatase (ALP) (U/L) activity. All analyses were performed using an automated system (Abbott Architect ci8200, Lake Forest, IL, USA).

### 2.9. Statistical Analysis

All data obtained from the study were analyzed using GraphPad Instat 3.10 (GraphPad Software, San Diego, CA, USA) (153). One-way analysis of variance (one-way ANOVA) was used to determine the differences between the experimental groups when looking at a single dependent variable. The Tukey–Kramer multiple test was applied after ANOVA for comparison of groups. All data were expressed as the mean standard error of the mean and a *p*-value less than 0.05 (*p* < 0.05) was considered statistically significant.

## 3. Results

### 3.1. Macroscopic and Microscopic Evaluations of Gastric Tissues

**Figure 1:**
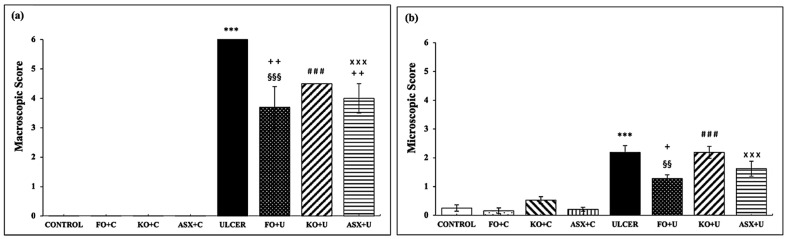
(**a**) macroscopic score, (**b**) microscopic score. C: Control, FO: Fish oil, KO: Krill oil, ASX: Astaxanthin, U: Ulcer. According to the statistical evaluation results, *** *p* < 0.001 compared to the control group; + *p* < 0.05 compared to the ulcer group; ++ *p* < 0.01 compared to the ulcer group; §§ *p* < 0.01 compared to the FO + C group; §§§ *p* < 0.001 compared to FO + C; ### *p* < 0.001; Compared to KO + C; ××× *p* < 0.001 compared to ASX + C group.

Oral gavage application of ethanol to rats resulted in an extensive ulcer-inducing gastric lesion (6.0 ± 0.0 mm), where the macroscopic score markedly exceeded that of the control group (0.0 ± 0.0 mm), *p* < 0.001. Figure 1a shows that supplementation with KO (KO + C), FO (FO + C), and ASX (ASX + C) did not change the gastric mucosa in the absence of ethanol exposure. Dietary supplementation with FO and ASX reduced the ulcer index significantly in the FO + U group (3.7 ± 0.7 mm) and the ASX + U (4.0 ± 0.5 mm) group, respectively, relative to the ulcer group (*p* < 0.01). However, the macroscopic scores between the KO-supplemented ulcer group (KO + U; 4.5 ± 0.5 mm) and the ulcer group (*p* > 0.05) lacked significant differences.

When gastric tissues were scored microscopically for surface epithelial desquamation, hemorrhage, glandular damage, and inflammatory cell infiltration, the results were compatible with the macroscopic scoring. Similarly, the microscopic damage scores in gastric tissues of the ethanol-induced ulcer-treated group (2.2 ± 0.2) significantly surpassed those of the control group (0.2 ± 0.1), *p* < 0.001 (Figure 1b). KO and ASX supplementation before ulcer treatment in the KO + U (2.2 ± 0.2) and ASX + U (1.6 ± 0.3) groups did not produce significant changes (*p* > 0.05). However, pretreatment with FO significantly reduced microscopic ulcer scores in the FO + U group (1.3 ± 0.1) relative to the ulcer group (*p* < 0.05).

**Figure 2:**
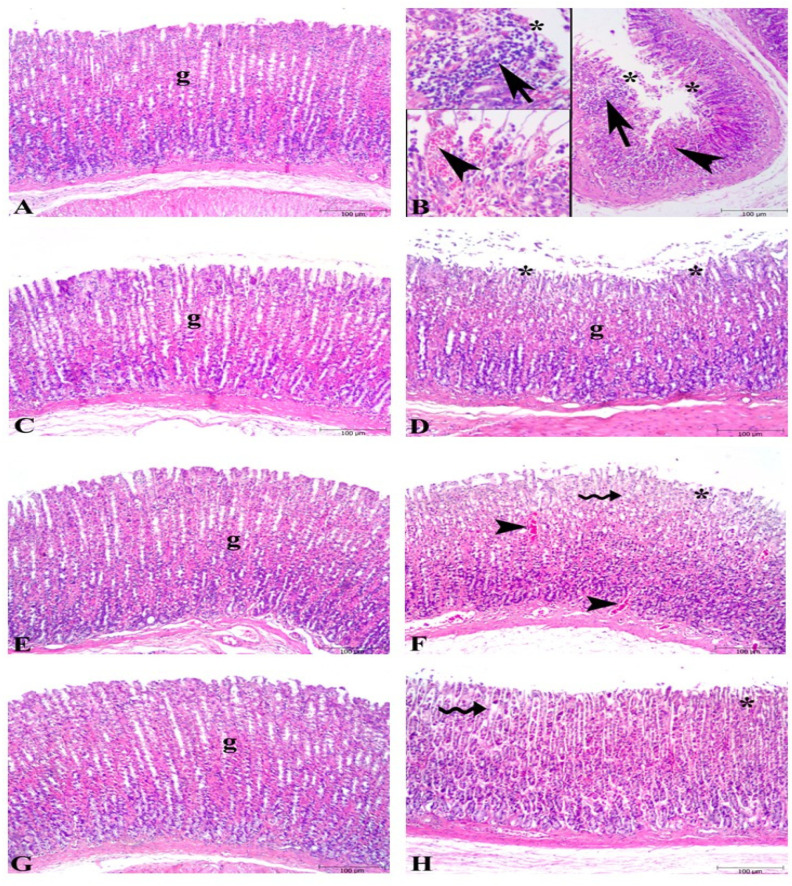
Photomicrographs of the gastric tissue in the experimental groups. (**A**) Control group, regular stomach mucosa with surface epithelium and glandular cells (g). (**B**) Severe damage to surface epithelium (*), hemorrhage (arrowhead), and inflammatory cell infiltration (arrow) in untreated ulcer groups. (**C**) Fish oil-treated control group, regular glandular cells (g) and surface epithelium. (**D**) Fish oil-treated ulcer group micrograph demonstrates nearly regular glandular cells (g) and mild degeneration in surface epithelium (*). (**E**) Krill oil-treated control group, regular glandular cells (g) in the stomach mucosa. (**F**) Krill oil-treated ulcer group, significant damage in surface epithelium (*), degeneration in glandular cells (wavy arrow), and hemorrhage (arrowhead). (**G**) Astaxanthin-treated control group, regular glandular cells (g) in the gastric mucosa. (**H**) Astaxanthin-treated ulcer group, mild dilated gastric glands (wavy arrow), and minimal degeneration at surface epithelium. (Hematoxylin and eosin staining, bars: 100 µm).

Microscopic examination of gastric tissues showed severe damage to surface epithelium, hemorrhage, and inflammatory cell infiltration in the ulcer groups (Figure 2), whereas the control group exhibited a regular stomach mucosa with surface epithelium and glandular cells. The KO-treated ulcer group (KO + U) showed significant damage in surface epithelium, degeneration in glandular cells, and hemorrhage like the ulcer group. Minimal degeneration at the surface epithelium and mild dilated glandular gastric glands were found in the FO- and ASX-supplemented ulcer groups (FO + U and ASX + U).

### 3.2. MDA–GSH Levels and MPO Activities in Gastric Tissues

**Figure 3:**
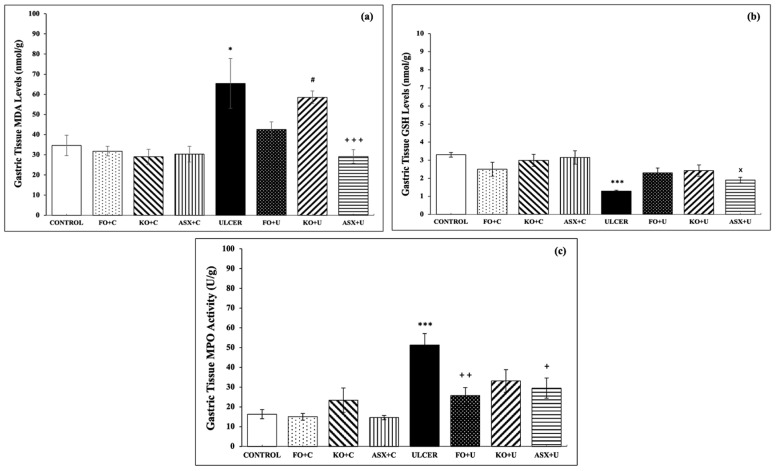
(**a**) malondialdehyde (MDA) levels, (**b**) glutathione (GSH) levels, (**c**) myeloperoxidase (MPO) activity. C: Control, FO: Fish oil, KO: Krill oil, ASX: Astaxanthin, U: Ulcer. According to the statistical evaluation results, * *p* < 0.05, compared to the control group; *** *p* < 0.001, compared to the control group; + *p* < 0.05, compared to the ulcer group; ++ *p* < 0.01 compared to ulcer group; +++ *p* < 0.001, compared with the ulcer group; # *p* < 0.05, compared to the KO + C group; × *p* < 0.05 compared to the ASX + C group.

The control group showed markedly lower levels of MDA, a specific biomarker for determining lipid peroxidation, than the ethanol-induced ulcer group (34.7 ± 5.1 nmol/g tissue vs. 65.4 ± 12.4 nmol/g tissue; *p* < 0.05). FO and KO administration to the ulcer group (FO + U: 42.6 ± 3.8 nmol/g tissue and KO + U: 58.6 ± 3.1 nmol/g tissue) reduced MDA formation slightly, but the results were not significant, *p* > 0.05. ASX supplementation markedly decreased MDA levels in gastric tissues of the ethanol-induced ulcer group (ASX + U: 42.6 ± 3.8 nmol/g tissue), *p* < 0.001 (Figure 3a).

As expected, the ethanol-induced ulcer groups exhibited decreased gastric tissue GSH (3.3 ± 0.1 μmol/g tissue), a powerful antioxidant molecule, relative to the control group (1.3 ± 0.1 μmol/g tissue), *p* < 0.001. Supplementation with FO, KO, or ASX increased the intracellular antioxidant content of GSH, but the results were not significant (*p* > 0.05; Figure 3b).

MPO activity, which is accepted as an indicator of neutrophil infiltration of the inflamed tissue, was significantly higher in gastric tissues of the ethanol-induced ulcer group than in the control group (51.3 ± 5.8 U/g tissue vs. 16.3 ± 2.3 U/g tissue; *p* < 0.001). KO supplementation to the KO + U group reduced the MPO activity, but the results were not significant (33.1 ± 5.8 U/g tissue; *p* > 0.05). FO supplementation to the FO + U group (25.7 ± 4.1 U/g tissue; *p* < 0.01) and ASX supplementation to the ASX + U group (29.5 ± 5.1 U/g tissue; *p* < 0.05) decreased MPO activity in gastric tissue samples significantly (Figure 3c).

### 3.3. Chemiluminescence Levels (CL) in Gastric Tissues

The CL method can be used for ROS measurements via enhancers. Hydroxyl radicals, H_2_O_2_, hydroperoxyl, and hypochlorite molecules can be measured as a sum of luminol-enhanced CL. As listed in Table 2, the gastric tissue CL levels of the ethanol-induced ulcer group (122.3 ± 4.2 rlu/mg) markedly exceeded those of the control group (74.1 ± 2.0 rlu/mg); *p* < 0.001. FO, KO, and ASX supplementation to ulcer groups (FO + U, KO + U, and ASX + U) reduced the luminol-enhanced ROS measurement significantly. Furthermore, lucigenin-enhanced CL measurement results were increased in gastric tissues of the ulcer group relative to the control group, *p* < 0.01. Supplementation to the FO + U, KO + U, and ASX + U groups significantly decreased superoxide radical formation compared to the ethanol-induced ulcer group (*p* < 0.001 for all groups). NO release and peroxynitrite levels in gastric tissues of the ulcer group significantly exceeded those of the control group (147.5 ± 10.6 rlu/mg vs. 62.4 ± 2.4 rlu/mg; *p* < 0.001 and 125.4 ± 4.7 rlu/mg vs. 60.1 ± 1.3 rlu/mg; *p* < 0.001; respectively). The NO and peroxynitrite levels in the FO + U, KO + U, and ASX + U groups were significantly decreased relative to the ulcer group (Table 2).

### 3.4. Microscopic Evaluations of Hepatic Tissues

**Figure 4:**
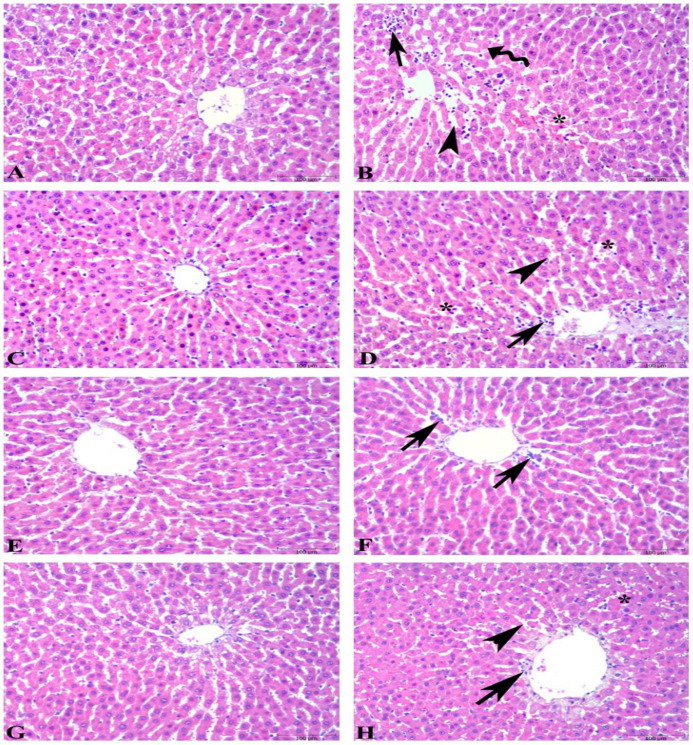
Photomicrographs of the liver tissue in the experimental groups. (**A**) Control group, regular histology of liver tissue. (**B**) Ulcer group, degenerated hepatocytes (wavy arrow), inflammatory cell infiltration (arrow), dilatation (arowhead), and vascular congestion in sinusoids (*). (**C**) Fish oil-treated control group, regular histology of liver tissue. (**D**) Fish oil-treated ulcer group, degeneration findings similar to ulcer group: inflammatory cell infiltration (arrow), dilatation in sinusoids (arowhead), and vascular congestion (*). (**E**) Krill oil-treated control group, regular hepatocytes and central vein. (**F**) Krill oil-treated ulcer group, regression in hepatocyte degeneration and decrease in inflammatory cell infiltration (arrow). (**G**) Astaxanthin-treated control group, regular hepatocytes and central vein. (**H**) Astaxanthin-treated ulcer group, decrease in sinusoidal congestion (*), sinusoidal dilatation (arrowhead), and inflammatory cell infiltration (arrow). (Hematoxylin and eosin staining, bars: 100 µm).

Microscopic scoring of hepatic tissues showed a significant increase in the ethanol-induced ulcer group compared to the control group (6.4 ± 0.8 vs. 1.0 ± 0.3, *p* < 0.001). However, in the ulcer-induced supplementation groups, the microscopic scoring index was significantly reduced compared to the ulcer group. The microscopic scores were as follows: FO + U group 4.1 ± 0.4 (*p* > 0.05), KO + U group 3.7 ± 0.5 (*p* < 0.01), and ASX + U group 1.3 ± 0.6 (*p* < 0.001). The KO + C, FO + C, and ASX + C groups had low microscopic scores of 1.9 ± 0.3, 1.3 ± 0.2, and 2.2 ± 0.4, respectively. Microscopic evaluation shows degenerated hepatocytes, inflammatory cell infiltration, dilatation, and vascular congestion in sinusoids of hepatic tissues in the ulcer and FO + U groups. However, in the KO + U group, regression in hepatocyte degeneration and decrease in inflammatory cell infiltration occurred. ASX supplementation decreased sinusoidal congestion in the ASX + U group. Control, KO + C, FO + C, and ASX + C groups exhibited regular histology of liver tissues (Figure 4).

### 3.5. MDA–GSH Levels and MPO Activities in Hepatic Tissues

The MDA and GSH levels did not change significantly in hepatic tissues of the experimental groups (*p* > 0.05). MPO activity in hepatic tissues increased in the ethanol-induced ulcer group, but the results were not statistically significant (*p* > 0.05).

### 3.6. Chemiluminescence Levels in Hepatic Tissues

Luminol- and lucigenin-enhanced CL measurements did not change in hepatic tissues of the experimental groups (*p* > 0.05; Table 3). Nevertheless, NO release and peroxynitrite levels were significantly higher in the ethanol-induced ulcer group than in the control group. Supplementation with FO, KO, and ASX significantly reduced NO release and peroxynitrite formation in the FO + U, KO + U, and ASX + U groups.

### 3.7. Serum Lipids and Liver Function Tests in Serum Samples

As listed in Table 4, FO treatment significantly reduced the lipid parameters, such as total cholesterol, LDL, and HDL levels, in the serum samples of the rats (*p* < 0.001). KO treatment also reduced these serum lipids, but the results were not statistically significant (*p* > 0.05). ASX supplementation did not affect serum lipids. Liver functions remained unaltered after supplementation with FO, KO, and ASX relative to the groups without supplementation (C + U groups).

## 4. Discussion

The study findings reveal that supplementation with FO, KO, and ASX provides protective, antioxidant, and anti-inflammatory effects at different levels in rats exposed to ethanol-induced ulcers.

In the present study, the macroscopic data obtained by examining bleeding spots and punctate erosions showed a significant decrease with FO and ASX application, while KO application decreased the lesions but was not statistically significant. Histopathologic examinations showed that FO treatment was more effective in preventing lesion formation than the KO and ASX treatments. Corroborating this study, research using an experimental ethanol-induced ulcer model revealed that ulcerated areas decreased, mucus content increased, and protection against ulceration improved by 67.46% in the group receiving *n*-3 supplementation via oral gavage for 14 days [40]. Bhattacharya et al. examined the effects of FO supplementation at different doses (50, 100, and 200 mg/kg) for 5 days on gastric ulcers and showed that even the lowest dose of FO decreased the ulcer index and gastric acid secretion [41]. Another study found that FO supplementation for 21 days before aspirin intake did not significantly reduce PGE2 levels or gastric damage in healthy adults [42]. While our study observed that FO administration had beneficial effects on gastric ulcerations, the effect of FO supplementation was not observed at the level of tissue oxidative stress parameters. In a dextran sulfate sodium-induced ulcerative colitis model, 5% KO supplementation for 4 weeks maintained colon length and significantly improved inflammation-related IL and PG levels in rats, hence suppressing NF-kB activity and cytokine production [23]. Zhou et al. reported that KO supplementation in mice with dextran sodium sulfate-induced ulcerative colitis for 21 days at a high dose (0.5 g/kg) preserved colonic mucosal integrity, whereas this effect was not observed at a low dose (0.25 g/kg) [43]. In this study, KO did not show the expected protective effect. Similar to the study by Zhou et al., even though the dose administered in the present study was sufficient, the duration of administration may have been insufficient [43]. In an ethanol-induced experimental ulcer model, 500 μg/kg ASX supplementation for 21 days protected the stomach mucin layer by 67%, inhibited acid formation by inhibiting the H^+^/K^+^-ATPase enzyme, and prevented ulcer formation [44]. ASX supplementation at various doses showed a protective effect against gastric ulcer formation [28,45]. In patients diagnosed with *H. pylori*, ASX supplementation for 8 weeks did not change inflammatory cytokine levels but enhanced humoral immunity (through upregulation of CD4 expression and downregulation of CD8 expression) [46]. The different results in these studies show that the optimal doses and application times of FO, KO, and ASX in experimental animal studies remain unclarified.

The stomach and upper gastrointestinal tract are the main sites of ethanol metabolism. Ethanol metabolism generates free radicals, especially superoxide radicals, and promotes lipid peroxidation. Additionally, gastric mucosal injury after ethanol exposure stimulates neutrophils and increases oxygen radicals. Neutrophil infiltration is a major result of gastric injury. Both types of damage can be assessed by measuring tissue-associated MDA levels and MPO activity, respectively [47]. In this study, ethanol-induced gastric injury significantly increased MDA levels and MPO activity in the ulcer group, and KO or FO supplementation to the ulcer groups failed to significantly reduce lipid peroxidation occurrence. However, FO supplementation significantly reduced neutrophil-related MPO activity in the FO + U group. Shaaban et al. showed that oral omega-3 supplementation at different doses (75 and 150 mg/kg) for 12 weeks markedly reduced serum MDA levels in rats with hepatic fibrosis [48]. In a TNBS-induced colitis model, rectal administration of FO improved serum and tissue MDA levels but did not result in a significant change [49]. In another study, FO administration with a high-fat diet for 8 weeks did not change serum MDA levels in hypercholesterolemic rats [50]. In a gentamicin-mediated nephrotoxicity model, KO supplementation had no effect on tissue MDA and total antioxidant capacity but caused changes at the histopathological level [51].

KO supplementation decreased serum MDA levels in rats with ischemia-reperfusion injury [52]. KO can inhibit lipid oxidation [53,54] and its supplementation in healthy adults (2 g/day for 6 weeks) did not change plasma TBARS, IL-6, IL-7, and IFNγ levels [55]. As seen in the literature, the effects of FO and KO on lipid peroxidation vary. Furthermore, FO supplementation can reduce gastric inflammation after ethanol-induced ulcer formation. Experimental studies have shown that FO supplementation reduced inflammatory cytokines, such as TNF-α, IL-1β, IL-6, MIP-1α, MCP-1, and leukotriene B_4_ [56], and attenuated macrophage infiltration, apoptosis, and NO content [57]. In this study, although KO was expected to be superior to FO and ASX supplements due to its EPA-DHA and ASX content, this effect did not manifest in MDA and GSH levels or MPO activity. As seen in the literature, different results have been obtained. This suggests that the effects of duration of KO application and application dose on tissues occur through different molecular pathways. Ulven et al. concluded that FO upregulated the cholesterol synthesis pathway and this difference in biological effect may be caused by the various structures of phospholipids in KO and triglyceride in FO [19].

ASX supplementation in various ulcer models yielded increased antioxidant enzyme activities—such as catalase (CAT), superoxide dismutase (SOD), and glutathione peroxidase (GPX)—and decreased lipid peroxidation levels in the ulcer group [28,44,45,58]. The ASX molecule has a structure containing hydroxyl and keto groups responsible for high antioxidant properties on each ionone ring. Therefore, it inhibits radicals in the cell membrane and scavenges radicals via their terminal rings in the outer and inner parts of the cell membrane. In addition, the oxyfunctional group in carotenoids has high antioxidant activity [59,60]. In the present study, ASX inhibited ethanol-induced ulcer oxidative stress due to its strong antioxidant capacity and showed a protective effect against lesions. This is evidenced by the decreased tissue MDA and MPO values and increased GSH levels in the ASX-treated ulcer-induced group. The protective effect of ASX against gastric lesions may be due to its ability to inhibit the H^+^/K^+^-ATPase enzyme, which markedly contributes to ulcer pathogenesis [41].

GSH is an antioxidant molecule that forms spontaneously within cells. It plays many biological roles, including DNA and protein synthesis, regulation of enzyme activities, and intracellular and extracellular transport, and it is also closely associated with the antioxidant system [61]. In the present study, intracellular GSH levels decreased in the ulcer group but did not change significantly in rats receiving KO, FO, and ASX supplementation. In our ethanol-induced ulcer model, the experimental animals were sacrificed 1 h after ethanol administration, which may not be sufficient for glutathione formation [62,63]. Actually, the decrease of GSH levels in gastric tissues may be due to its consumption during the oxidative stress induced by alcohol. According to our findings, this result was similar to the study by İpek et al. [32]. In the same study, it was shown that the application of metformin, an antidiabetic drug, to rats increased SOD enzyme activity, which catalyzes the conversion of superoxide anion to H_2_O_2_, in gastric samples. In our study, intensive FO, KO, and ASX administration before ulcer formation did not have a stimulating effect on gastric tissue to increase GSH synthesis. However, in various inflammatory settings, FO, KO, and ASX were reported to have antioxidant properties beyond their effects. In an experimentally developed cold restraint stress model, it has been demonstrated that FO application reduces SOD enzyme activity in a dose-dependent manner, while simultaneously increasing the activities of CAT and GPX enzymes [41]. Among these enzymes, CAT is one of the key enzymes that catalyzes the conversion of H_2_O_2_ to water, and GPX aims to reduce oxidative stress damage by forming GSH to oxidized GSSG. Similarly, it has been reported that high doses of KO in a different ethanol-induced ulcer model decreased SOD enzyme activity [64]. A comprehensive experimental ulcer study examining antioxidant enzyme activities has shown that ASX application increases SOD, CAT, and GPX enzyme activities in a dose-dependent manner [44]. These studies indicate that GSH formation and the GSH-mediated enzyme cycle are inhibited and/or consumed with ulcer formation but can be partially restored with FO, KO, or ASX application. Although our study does not examine GSH-mediated enzyme activities, but the observed trend of increased GSH levels in the gastric tissue of groups treated with FO, KO, and ASX, along with the reductions in lipid peroxidation, can be interpreted as an attempt to restore the GSH-mediated enzymatic mechanism.

In this study, CL analysis revealed that mitochondrial free radicals associated with both inflammation and ischemic damage increased in the ethanol-induced experimental ulcer model, indicating that ROS and reactive nitrogen species (RNS) increased after ethanol-induced ulcer formation. In addition, FO, KO, and ASX supplementation to ulcer-created gastric tissues reduced all species of ROS and RNS. Şehirli et al. studied the effects of α-lipoic acid on ethanol-induced gastric ulcers and found that ethanol administration increased luminol- and lucigenin-mediated CL levels. This finding, corroborating ours, supports the idea that ethanol-induced gastric damage generates toxic oxygen metabolites, such as hydroxyl radicals, H_2_O_2_, hydroperoxyl, hypochlorite, and superoxide radicals [47]. Luminol- and lucigenin-mediated ROS formation in the gastric tissues of rats treated with FO and KO were significantly decreased relative to the non-supplemented ulcer group. ASX administration prevented ROS formation caused by ethanol-mediated ulcer damage with its strong antioxidant effect and reduced ROS formation to the basal level. Notably, ethanol rapidly passes through the gastric mucosa, causing endothelial damage in blood vessels through membrane injury and increasing mucosal permeability. Vascular and microvascular changes accelerate the formation of gastric ulcers. ASX pretreatment increases mucus production by blocking the H^+^/K^+^-ATPase proton pump, thereby protecting the gastric mucosal layer from free radical damage and gastric acid secretion [65]. In an experimental ethanol/HCl ulcer model where a single dose (30 or 100 mg/kg BW; 1 h) of ASX was applied, it was shown that the loss of epithelial cells in gastric tissue decreased [58]. In a similar experimental study on a pancreatitis model in rats, Gürler et al. found that a single dose (40 mg/kg BW; 1 h) of ASX administered orogastrically decreased luminol- and lucigenin-enhanced ROS formation and increased GSH content [66]. In another experimental ulcer model, ASX supplementation had a protective effect against ulcers due to its antioxidant properties and provided 23 times more LOX enzyme inhibition compared to PPI omeprazole [44], indicating its powerful anti-inflammatory effect. A study exploring various hydrophilic and lipophilic antioxidants implicated ASX as the molecule with the strongest singlet oxygen scavenging activity [67]. Owing to its chemical structure, ASX is more stable than many other antioxidant molecules, neutralizes ROS in mitochondria, and protects the cell membrane against oxidative stress damage [68,69]. ASX localizes not only to the cell membrane but also to the mitochondrial membrane, where it affects cytochrome c and pro-apoptotic mechanisms, preventing the increase of free radicals. Moreover, through an indirect pathway, it activates antioxidant signaling pathways, regulating mitochondrial redox status and maintaining membrane integrity [65]. In this study, ROS levels were significantly decreased thanks to the strong antioxidant potential of ASX, in support of the literature.

Nitric oxide is synthesized by nitric oxide synthase (NOS) in three different ways. Endothelial NOS (eNOS)-mediated NO causes vasodilation of endothelial cells, and neuronal NOS (nNOS)-mediated NO acts as a second messenger molecule in neurons. Inducible NOS (iNOS)-mediated NO is synthesized from leukocytes to promote inflammation. Mitochondrial superoxide radicals are the most important product of mitochondrial redox state change, especially in ischemia and inflammation. Under severely toxic conditions, the peroxynitrite radical, a more toxic molecule, is formed in the presence of superoxide radicals and NO [70]. In the present study, both NO and peroxynitrite radicals increased in the gastric tissue of rats with ethanol-induced ulceration. Histopathologic examination of the ulcers revealed cell infiltration, migration of leukocytes to the lesion areas, and increased inflammation. Our findings suggest that NO release is mostly mediated by leukocyte-mediated iNOS. In an acetic acid-induced colitis model, acetic acid administration increased luminol, lucigenin, NO, and peroxynitrite CL levels [71].

In the present study, the animals were euthanized 1 h after the experimental ulcer model was established, and the effect of ethanol on liver function tests was examined within this 1 h period. As a result of the regular use of KO, FO, and ASX, changes that may occur in triglyceride and fat metabolism can be seen in the blood. Therefore, the supplementation and ulcer groups were evaluated together. KO and FO treatments decreased serum total cholesterol, triglyceride, and LDL levels relative to the non-supplemented groups. However, while the FO administration results were significant, those of KO administration were not. Vigerust et al. found that in C57BL/6hTNF-α transgenic mice, KO was superior to FO in reducing triacylglycerol levels. Although the difference was not statistically significant, total cholesterol, HDL cholesterol, and LDL cholesterol levels were found to be lower in the group receiving the FO-rich diet [72]. In a double-blind, crossover, placebo-controlled, randomized trial, Ramprasath et al. found that KO supplementation for 4 weeks in 24 healthy adults increased total and LDL cholesterol levels, while serum triglyceride and HDL cholesterol levels did not change with either KO or FO treatment—findings that may have resulted from the participants being normolipidemic individuals [73]. Some studies have uncovered that KO administration in adults yielded hypolipidemic activity, with this effect being pronounced in participants with hyperlipidemia [74,75]. Tillander et al. administered C57BL/6J mice with a diet containing high levels of fat (24% fat), FO (5.8% FO), or KO (5.7% KO) for 6 weeks. Plasma total cholesterol, triacylglycerol, and phospholipids were significantly decreased by FO administration, while KO decreased non-esterified fatty acids. Further analysis revealed that the effects of FO and KO on lipid metabolism were mediated by different pathways. While FO inhibited PPAR-α activation in the liver and intestine, KO administration decreased the expression and activity of fatty acid synthase, acetyl-CoA carboxylase, and HMG-CoA reductase enzymes. PPAR-α activation increases the hepatic expression of lipogenic genes, with a simultaneous increase in fatty acid synthesis and TAG accumulation. In addition this study found a decrease in the gene expression of proteins involved in cholesterol and fatty acid synthesis due to KO administration [76]. These differences in effect may be attributed to differences in the structure of *n*-3 fatty acids found in KO and FO [73,76]. Current findings in the literature support the results of this study.

Liver functional enzymes remained unchanged after four-week treatment with FO, KO, and ASX supplementation. Sistilli et al. found that KO supplementation reduced ALT levels and hepatic steatosis in mice fed a diet containing 30% triglyceride form *n*-3 (FO) and KO for 24 weeks. However, no significant difference was observed in AST levels [77]. Hwang et al. found that KO administration together with metformin decreased ALT, AST, and ALP levels and provided blood glucose homeostasis in obese mice fed a high-fat diet for 12 weeks [78]. These studies were conducted on subjects with hepatic damage or fatty deposits. Notably, in the present study, KO supplementation increased liver AST enzyme activity, which is bound to the mitochondria of hepatocytes. This may be explained by the effects of KO on the expression of mitochondrial enzyme activities [76].

## 5. Conclusions

Studies have shown that KO has beneficial effects in protecting against various diseases due to its rich *n*-3 content and bioactive components. To our knowledge, no studies in the literature have compared the protective effects of KO, FO, and ASX against gastric ulcers. In the present study, contrary to expectations, daily feed consumption with KO supplementation for 4 weeks at a rate of 2.5% (*v*/*w*) via the orogastric route was not superior to FO and ASX in protecting against ethanol-induced ulcers in rats. Ethanol-induced treatment increased oxidative damage (as seen in the MDA and CL analysis results) and inflammation (evidenced by MPO activity and microscopic evaluation) while reducing antioxidant (GSH level) capacity. ASX inhibited lipid peroxidation and neutrophil infiltration caused by oxidative stress and protected gastric tissue against ROS-induced damage. Although improvements were observed in some parameters examined upon KO application, similar effects were also produced by FO.

## Figures and Tables

**Table 1:** Macroscopic lesion scoring of gastric tissue.

Lesion Size	Scoring
No damage	0
Presence of blood in the lumen	1
Point-type erosions;	2
1–5 small erosions (<2 mm);	3
>5 small erosions;	4
Large erosions (>2 mm);	5
>3 large erosions	6

**Table 2:** Gastric tissue CL levels.

Gastric Tissue CL Values	C	FO + C	KO + C	ASX + C	U	FO + U	KO + U	ASX + U
Luminol CL (rlu/mg)	74.1 ± 2.0	53.8 ± 2.4	64.9 ± 5.6	28.2 ± 1.3	122.3 ± 4.2 ***	102.9 ± 2.8 ^++^	94.2 ± 3.2 ^+++^	33.9 ± 3.1 ^+++^
Lucigenin CL (rlu/mg)	54.3 ± 1.0	55.1 ± 6.5	52.3 ± 4.6	17.3 ± 1.3	140.3 ± 14.0 **	91.5 ± 4.7 ^+++^	99.0 ± 26.9 ^+++^	31.1 ± 1.0 ^+++^
NO Release (rlu/mg)	62.4 ± 2.4	51.9 ± 6.3	49.6 ± 2.6	24.3 ± 2.3	147.5 ± 10.6 ***	97.6 ± 3.7 ^+++^	89.1 ± 5.1 ^+++^	24.6 ± 3.3 ^+++^
Peroxynitrite Release (rlu/mg)	60.1 ± 1.3	36.2 ± 1.2	57.6 ± 4.7	17.8 ± 0.3	125.4 ± 4.7 ***	97.8 ± 1.6 ^+++^	90.5 ± 4.5 ^+++^	21.6 ± 1.0 ^+++^

C: Control, FO: Fish oil, KO: Krill oil, ASX: Astaxanthin, U: Ulcer. According to the results of statistical evaluation, ** *p* < 0.01, compared with control group; *** *p* < 0.001, compared with control group; ++ *p* < 0.01 compared to ulcer group; +++ *p* < 0.001, compared with ulcer group.

**Table 3:** Liver tissue CL levels.

Liver Tissue CL Values	C	FO + C	KO + C	ASX + C	U	FO + U	KO + U	ASX + U
Luminol CL (rlu/mg)	20.4 ± 1.4	19.7 ± 1.0	18.0 ± 0.7	20.3 ± 1.0	23.6 ± 2.9	17.3 ± 1.1	18.6 ± 1.7	19.3 ± 2.5
Lucigenin CL (rlu/mg)	19.5 ± 1.6	20.0 ± 3.2	19.3 ± 1.1	17.9 ± 0.9	21.6 ± 0.9	17.7 ± 0.7	21.3 ± 1.0	20.3 ± 0.8
NO Release (rlu/mg)	15.4 ± 0.6	17.6 ± 0.6	17.8 ± 1.1	14.6 ± 0.5	22.1 ± 1.2 ***	18.9 ± 0.8	17.5 ± 1.0 ^++^	14.6 ± 0.3 ^+++^
Peroxynitrite Release (rlu/mg)	15.8 ± 0.5	13.4 ± 0.5	17.8 ± 0.9	12.8 ± 0.6	19.8 ± 1.0 **	15.3 ± 0.9 ^+++^	16.1 ± 0.5 ^+^	14.7 ± 0.5 ^+++^

C: Control, FO: Fish oil, KO: Krill oil, ASX: Astaxanthin, U: Ulcer. According to the results of statistical evaluation, ** *p* < 0.01, compared with control group; *** *p* < 0.001, compared with control group; + *p* < 0.05, compared with ulcer group; ++ *p* < 0.01, compared with ulcer group; +++ *p* < 0.001, compared with ulcer group.

**Table 4:** Results of serum lipids and liver function tests in serum samples.

	C + U	FO	KO	ASX
Lipid Parameters				
Total Cholesterol (mg/dL)	54.8 ± 4.8	37.7 ± 2.2 ***	49.6 ± 2.4	52.3 ± 1.4
Triglyceride (mg/dL)	55.0 ± 4.4	40.1 ± 3.2	49.6 ± 5.4	59.9 ± 5.0
LDL cholesterol (mg/dL)	10.7 ± 1.2	6.2 ±0.5 ***	9.4 ± 0.7	10.6 ± 0.6
HDL cholesterol (mg/dL)	42.9 ± 4.1	28.9 ± 1.9 **	37.6 ± 1.9	40.3 ± 1.5
Liver Function Tests				
ALT (U/L)	40.2 ± 3.0	44.5 ± 3.3	41.8 ± 3.5	38.8 ± 2.3
AST (U/L)	88.2 ± 9.5	82.5 ± 3.3	119.2 ± 7.0 *	94.8 ± 8.9
ALP (U/L)	89.0 ± 5.0	90.9 ± 3.9	104.6 ± 6.7	62.7 ± 2.0 **

C: Control, U: Ulcer, FO: Fish oil, KO: Krill oil, ASX: Astaxanthin. According to the statistical evaluation results, *** *p* < 0.001, ** *p* < 0.01, * *p* < 0.05 compared with C + U group.

## Data Availability

The data obtained in this study are available from the corresponding author upon request.
